# Comparative genomics of Australian and international isolates of *Salmonella* Typhimurium: correlation of core genome evolution with CRISPR and prophage profiles

**DOI:** 10.1038/s41598-017-06079-1

**Published:** 2017-08-29

**Authors:** Songzhe Fu, Lester Hiley, Sophie Octavia, Mark M. Tanaka, Vitali Sintchenko, Ruiting Lan

**Affiliations:** 1 School of Biotechnology and Biomolecular Sciences, University of New South Wales, Sydney, New South Wales Australia; 2 Public Health Microbiology Laboratory, Forensic and Scientific Services, Queensland Department of Health, Brisbane, Queensland Australia; 3 Marie Bashir Institute for Infectious Diseases and Biosecurity, University of Sydney, Sydney, New South Wales Australia; 4 Centre for Infectious Diseases and Microbiology–Public Health, Institute of Clinical Pathology and Medical Research, Westmead Hospital, Sydney, New South Wales Australia

## Abstract

*Salmonella enterica* subsp *enterica* serovar Typhimurium (*S*. Typhimurium) is a serovar with broad host range. To determine the genomic diversity of *S*. Typhimurium, we sequenced 39 isolates (37 Australian and 2 UK isolates) representing 14 Repeats Groups (RGs) determined primarily by clustered regularly interspaced short palindromic repeats (CRISPR). Analysis of single nucleotide polymorphisms (SNPs) among the 39 isolates yielded an average of 1,232 SNPs per isolate, ranging from 128 SNPs to 11,339 SNPs relative to the reference strain LT2. Phylogenetic analysis of the 39 isolates together with 66 publicly available genomes divided the 105 isolates into five clades and 19 lineages, with the majority of the isolates belonging to clades I and II. The composition of CRISPR profiles correlated well with the lineages, showing progressive deletion and occasional duplication of spacers. Prophage genes contributed nearly a quarter of the *S*. Typhimurium accessory genome. Prophage profiles were found to be correlated with lineages and CRISPR profiles. Three new variants of HP2-like P2 prophage, several new variants of P22 prophage and a plasmid-like genomic island StmGI_0323 were found. This study presents evidence of horizontal transfer from other serovars or species and provides a broader understanding of the global genomic diversity of *S*. Typhimurium.

## Introduction

*Salmonella enterica* serovar Typhimurium is the most common *Salmonella* serovar causing foodborne infections in Australia and many other countries^1^. The phenotypic diversity of *S*. Typhimurium has been traditionally illustrated by the Anderson phage typing scheme with more than 200 phage types defined^2^. Since then, different molecular markers were used to assess its genetic diversity^3, 4^. *S*. Typhimurium is divided by multilocus sequence typing into more than 30 sequence types (STs). ST19 is the most prevalent ST internationally and the majority of the STs belong to the ST19 clonal complex^5^. By application of whole-genome-sequencing (WGS) and genomic analysis, our earlier study of six Australian *S*. Typhimurium strains from different phage types and 7 published genomes revealed three genomic clusters^6^. Hayden *et al*.^7^ analysed the genomic diversity of 35 US *S*. Typhimurium isolates together with 21 public genomes^7^ and found that the 56 *S*. Typhimurium strains could be divided into 3 clades and at least 10 lineages.

Genome sequencing showed that variation among *S*. Typhimurium strains was mainly due to the accumulation of single nucleotide polymorphisms (SNPs). The *S*. Typhimurium genome consists of a core genome of around 3,800 genes present in all strains and nearly 1,000 accessory genes which are variably present in one or more strains^4, 7^. The accessory genome contains mostly genes of prophages and genes of unknown function which have contributed to the genetic diversity of *S*. Typhimurium^3, 4^. However, the full depth of genomic diversity of *S*. Typhimurium remains to be explored.

Clustered regularly interspaced short palindromic repeats (CRISPR) belong to a family of unique repeat sequences and are possibly associated with adaptive resistance against invasive genetic elements such as phages. *Salmonella* generally possesses two CRISPR loci, which comprise conserved direct repeats separated by unique short sequences typically 32 bp long, called spacers^8, 9^. Variation in the spacer profiles of CRISPRs has been useful for subtyping *Salmonella* isolates^10^. Shariat *et al*.^11^ investigated CRISPR array composition in four major serovars, Enteritidis, Typhimurium, Newport and Heidelberg, and demonstrated serovar specificity of CRISPR array composition^11^. Other studies have also found that CRISPR variation is associated with serotype and sequence types, providing good phylogenetic signals for inferring strain relationships^8, 12, 13^. Hiley *et al*.^14^ performed CRISPR and variable number tandem repeat (VNTR) analyses on a diverse collection of 200 Australian *S*. Typhimurium isolates and 14 reference strains which separated them into 15 Repeats Groups (RGs)^14^. This presented an opportunity to apply genomic analysis to selected isolates from these RGs to capture a broad range of genetic diversity of *S*. Typhimurium.

In this study, we sequenced 39 strains, 37 of which were of Australian origin, representing 14 different RGs and we included in the analysis 66 publicly available *S*. Typhimurium genomes including 54 of international origins to provide a global overview of *S*. Typhimurium diversity. We constructed a core genome phylogeny for all 105 strains and examined the correlation of core genome relationships with CRIPSR array composition and prophage profiles.

## Results and Discussion

### Selection of *S*. Typhimurium isolates for genome sequencing

Strains were selected to represent 14 of the 15 RGs previously defined by Hiley *et al*.^14^ (Supplementary Figures S1 and Table S1). No RG15 strains were chosen for this study as only 2 RG15 strains were found at the time of study^14^. RG4, RG5, RG6, RG9, RG10 and RG12 had been further subtyped into 2 to 4 sub-categories based on one or more spacer differences in CRISPR1 and/or CRISPR2 so isolates from each sub-category were chosen. In all, 37 Australian and two UK *S*. Typhimurium isolates were selected for genome sequencing to represent the diversity of these 14 RGs (Table 1).

**Table 1:** List of *S*. Typhimurium isolates sequenced in this study.

Strain Name	Year of Collection	MLVA Euro	MLVA Aust Code	Phage Type
L1848	2001	02-11-12-09-212	03-13-13-10-523	29
L1849	2004	04-12-20-07-211	05-14-21-08-490	12a
L1850	2005	03-09-NA-NA-111	04-11-00-00-463	44
L1851	2006	04-14-11-NA-211	05-16-12-00-490	141
L1852	2006	02-10-10-09-212	03-12-11-10-523	135a
L1853	2006	04-22-12-08-211	05-24-13-09-490	104 L
L1854	2007	02-11-10-09-212	03-13-11-10-523	135
L1855	2007	03-13-08-08-211	04-15-09-09-490	197
L1856	2007	02-10-10-09-212	03-12-11-10-523	135a
L1857	2007	02-23-12-10-212	03-25-13-11-523	9
L1858	2007	04-14-14-11-211	05-16-15-12-490	193
L1859	2007	02-26-15-11-212	03-28-16-12-523	8
L1860	2007	03-12-14-14-311	04-14-15-15-517	12
L1861	2007	02-09-09-07-212	03-11-10-08-523	U302
L1862	2007	04-13-08-08-211	05-15-09-09-490	6
L1863	2007	03-17-10-15-210	04-19-11-16-457	179
L1864	2008	02-11-11-08-212	03-13-12-09-523	102
L1865	2008	02-10-11-09-212	03-12-12-10-523	135a
L1866	2008	03-14-12-NA-311	04-16-13-00-517	170
L1867	2008	02-09-09-05-212	03-11-10-06-523	29
L1868	2008	02-08-07-08-212	03-10-08-09-523	186
L1869	2008	02-08-07-08-212	03-10-08-09-523	44
L1870	2008	02-11-08-09-212	03-13-09-10-523	135
L1871	2008	02-15-15-12-212	03-17-16-13-523	126
L1872	2008	02-13-15-08-212	03-15-16-09-523	135
L1873	2008	02-20-09-07-212	03-22-10-08-523	135
L1874	2008	03-11-09-NA-211	04-13-10-00-490	193
L1875	2008	03-13-14-NA-211	04-15-15-00-490	170
L1876	2008	02-13-NA-NA-311	03-15-00-00-517	120
L1877	2009	02-11-10-09-212	03-13-11-10-523	3
L1878	2009	03-18-10-NA-111	04-20-11-00-463	41
L1879	2011	07-13-NA-NA-211	08-15-00-00-490	197
L1880	2011	02-12-10-10-212	03-14-11-11-523	135a
L1881	2011	02-07-06-11-212	03-09-07-12-523	170
L1882	2011	02-10-11-10-212	03-12-12-09-523	135a
L1883	2011	02-07-07-11-212	03-09-08-12-523	170
L818	2001	02-14-18-09-212	03-16-09-11-523	41
L825	2002	05-10-NA-09-211	06-12-00-10-490	193
L930	1997	02-08-07-08-212	03-10-08-09-523	44

An additional 66 strains, including 21 *Salmonella* reference collection A (SARA) strains^4^ and 45 other publicly available genomes (including 11 from Australia), were also included in the analysis (Supplementary Table S2). The CRISPR compositions of these strains were determined using genome data or by PCR sequencing. We found some strains had CRISPR1 or CRISPR2 composition similar to one RG but the other CRISPR composition was similar to another RG. We called them hybrid RGs and designated the genotype as RG CRISPR1/CRISPR2 to indicate the genotype difference in the two CRISPRs. For instance, strain SARA4 was a RG12D/10A hybrid. Genome sequencing statistics are listed in Table 2. The reads were assembled *de novo*. The number of contigs produced ranged from 214 to 378, with an average of 294 per genome. SNPs were discovered by mapping to the reference *S*. Typhimurium strain LT2 (NCBI GenBank Accession No. NC_003197). The number of SNPs ranged from 128 to 11,319 relative to the strain LT2 (Table 2).

**Table 2:** General features of strains sequenced in this study.

Strain Name	Total No. of reads	N50	Contig Number	Total Length (bp)	Coverage (%)	No. of non-synonymous SNPs	No. of synonymous SNPs	No. of Intergenic SNPs	No. of Indels	Total No. of SNPs
L1848	832,934	270,522	252	4,878,662	22	276	189	83	45	593
L1849	1,928,202	191,995	278	4,878,775	50	265	254	88	50	657
L1850	1,918,013	217,086	366	4,912,563	69	250	217	104	55	626
L1851	1,407,422	187,086	246	4,839,606	27	68	27	12	38	145
L1852	2,262,488	225,499	360	4,935,680	46	345	301	139	68	853
L1853	1,381,622	93,669	279	4,848,641	25	492	277	133	60	962
L1854	2,458,806	172,518	271	4,873,430	40	272	209	144	69	694
L1855	1,939,904	243,033	284	4,870,570	42	301	250	107	56	714
L1856	2,289,358	191,815	287	4,911,441	53	297	216	137	66	716
L1857	2,510,832	225,471	276	4,893,660	47	373	309	149	73	904
L1858	1,784,738	461,717	214	4,942,127	31	59	21	12	36	128
L1859	2,589,462	227,006	436	4,950,039	70	324	297	196	50	867
L1860	1,091,774	204,354	292	4,972,319	42	321	283	116	60	780
L1861	2,452,986	211,251	261	4,960,484	53	360	340	148	69	917
L1862	2,271,550	247,127	346	4,997,225	37	270	209	83	55	617
L1863	1,961,248	484,774	213	4,834,244	37	289	270	99	46	704
L1864	1,382,398	250,313	250	4,920,225	25	280	190	120	55	645
L1865	1,911,482	243,303	278	4,908,702	28	287	210	110	48	655
L1866	1,981,270	207,403	255	4,853,339	38	162	112	83	51	408
L1866	1,195,260	270,542	279	4,924,157	31	358	312	142	68	880
L1868	1,943,952	170,885	279	4,969,999	28	335	297	128	60	820
L1869	2,349,774	165,711	326	4,911,892	32	347	372	147	62	928
L1870	1,280,956	242,937	275	4,881,193	30	301	211	126	66	704
L1871	2,703,908	178,436	291	4,860,646	62	436	289	173	95	993
L1872	2,557,384	219,635	370	4,882,236	69	303	322	125	72	822
L1873	1,775,906	208,524	331	4,890,388	34	228	141	86	56	511
L1874	1,911,482	271,008	347	5,052,975	35	248	201	95	63	607
L1875	2,119,420	247,035	243	4,773,579	40	272	258	101	61	692
L1876	1,348,454	188,257	266	4,773,760	27	1,831	8,183	1,236	89	11,339
L1877	1,454,232	149,277	309	4,875,957	29	292	201	134	58	685
L1878	1,259,628	266,717	304	4,748,690	60	308	232	166	72	778
L1879	1,684,386	232,300	316	4,963,003	41	1,166	7,465	1,588	72	10,291
L1880	1,338,944	256,474	285	4,913,050	32	265	212	152	64	693
L1881	1,590,652	200,287	299	4,915,853	51	309	242	170	68	789
L1882	2,646,186	232,306	274	4,914,415	32	266	215	157	56	694
L1883	2,351,622	221,927	313	4,915,122	48	320	235	165	66	786
L818	1,327,194	257,038	280	4,892,270	24	431	275	157	92	955
L825	1,426,998	209,878	255	4,894,398	26	283	200	97	61	641
L930	1,038,048	179,836	378	4,924,303	22	350	297	142	70	859

### Core and accessory genomes of *S*. Typhimurium

Pan genome analysis showed that there were 8,849 genes, including 3,836 core genes, and 5,013 dispensable genes in *S*. Typhimurium. The core genome was smaller than in our previous report (3,846 genes)^4^ and the reports of Hayden *et al*. (3,910 genes)^7^ and Mather *et al*. (3,890 genes)^15^. This finding indicated that the current dataset had a strain coverage broad enough to achieve a stable core genome and adding more strains to the analysis would not substantially reduce the core genome size. Interestingly, prophage genes contributed up to 13.3% (1,175/8,849) of the pan genome and 23.4% (1,175/5,013) of the accessory genome, while plasmid and other mobile elements took up 13.3% (668/5,013) of the accessory genome. For the rest of the genes in the accessory genome, the majority (2,043 genes) encoded hypothetical proteins with currently unknown function.

### Genomic relationships and their correlation with Repeats Groups (RGs)

**Figure 1:**
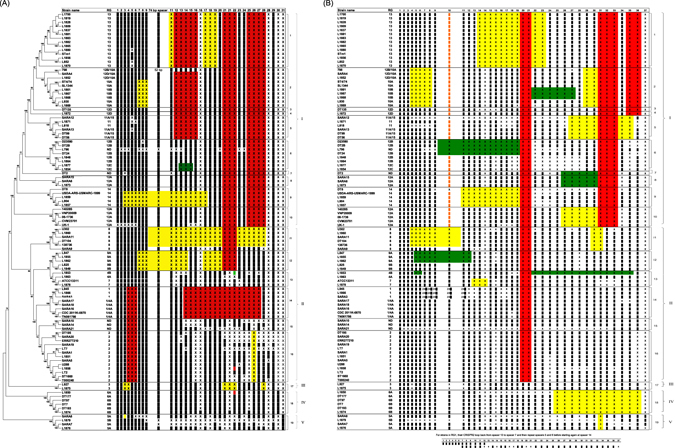
Phylogenetic relationship of 105 *S*. Typhimurium strains and its correlation with CRISPR1 (**A**) and CRISPR2 (**B**) patterns. The minimum evolution method was used to infer evolutionary relationships of the isolates based on their SNPs obtained from *S*. Typhimurium core genome. The Bootstrap was performed with 1,000 replicates. The bootstrap values (1,000 replicates, >50%) are shown next to the branches. The lineages (1 to 19) and clades (I to V) are indicated on the right side. CRISPR1 and CRISPR2 consist of 31 and 37 spacers respectively. The spacers are numbered in the order according to Table 6 from Fabre *et al*.^8^. The representation of CRISPR profiles is the presence (filled rectangular blocks) or absence (X) of the spacers in CRISPR1 and CRISPR2. In CRISPR1, the 74 bp spacer includes sp10. Sp22 contains a 6 bp tandem repeat; Green = 3 repeats, Black = 4 repeats, Red = 5 repeats, Blue = 6 repeats. In CRISPR2, sp10 variant that has one SNP is labelled in orange. The spacers lost in multiple RG, specific RG and sub-RG groups are highlighted in red, yellow and green, respectively.

A phylogeny of the 105 strains was constructed based on SNPs derived from the *S*. Typhimurium core genome^4^. The strains were separated into five clades and 19 lineages (Figs 1 and S1). Clade I contained RGs 10, 11, 12, 13 and 14 while Clade II contained RGs 1, 2, 4, 7, 8 and 9. Clades III, IV and V corresponded to RG3, RG6 and RG5 respectively. Clade V consisting of four strains L1876, L1879, SARA7 and SARA8 was the most distant clade being separated by more than 4,000 SNPs from the other clades as found in a previous study^4^. Clades III and IV were much closer to Clade II. Of the 19 lineages defined, 16 lineages contained more than one isolate and 3 lineages contained only one isolate. The strains falling within each lineage nearly always belonged to a single RG or combination of one RG and a CRISPR hybrid related to that RG and for the most part all the strains belonging to an RG fell into a single lineage. Exceptions were RG12A strains which were split into two lineages (lineages 10 and 8), separated by the RG14 lineage, and lineage 13 which contained strains belonging to RG4 and RG7 even though they are distinctly different by both CRISPR and VNTR profiles^14^ (Fig. 1).

Genomic typing resolved the phylogenetic relationships between RGs that were not clearly evident from the CRISPR profiles. Thus RG12D was the likely precursor for both RG10 and RG13 lineages. There was a loss of spacers from the RG12D CRISPR arrays specific to each of the RG10 and RG13 lineages. Three strains SARA10, SARA14 and SARA21 that could not be assigned to any recognised RG formed a separate lineage between RG1 and RG2.

Strains with hybrid RGs were genomically clustered with other strains which had the same or similar CRISPR1 RG or CRISPR2 RG. Five strains (i.e. TN061786, CDC 2011K-0870, SARA16, SARA17 and SARA18) shared similar CRISPR1 profiles with RG1 strains but their CRISPR2 profiles were like those in RG4A (RG1/4A in Fig. 1). They fell into the same lineage as RG1. Four strains, SARA12, SARA13, DT56 and DT99, were RG11A/15 hybrids and clustered with isolates belonging to RG11 genomically. SARA4 and L1852 were RG12D/10A hybrids which clustered with isolates belonging to RG10A. Likewise, strain 798 (NC _017046) which was a RG12B/10A hybrid was also genomically clustered with RG10A. These hybrids suggest that recombination has substituted CRISPR1 or CRISPR2 sequences of the respective strains in their common ancestors.

Our study revealed a higher genomic diversity of *S*. Typhimurium than that reported by Hayden *et al*.^7^, in which 10 lineages belonging to 3 clades were found among 35 sequenced US *S*. Typhimurium isolates and 21 published genomes. Examination of representative isolates in each lineage from Hayden *et al*.^7^ showed that only RG1, RG2, RG8, RG10, RG11A/15 and RG12 genotypes were included in their study, leaving an under-representation of the genomic diversity. Although genomes belonging to RG1, RG1/4A, RG3, RG5, RG6, RG9A, RG11A/15, RG12, RG13 and RG14, were also included in previous genome-sequencing studies^4, 6, 16, 17^, ours is the first such comprehensive study to also include RG4, RG7 and RG9B genomes. Further analysis of other published genomes did not uncover more RGs, suggesting that isolates included in this set represented the spectrum of RG diversity available to date (Table S3).

Comparison of Australian isolates and international isolates with published genomes showed that most RGs including RG1, RG2, RG5, RG6A and B, RG8, RG10A and B, RG11A and B, RG12A, B, C and D and RG14, contained isolates from Australia and other parts of the world, suggesting wide distribution of these RGs. Three RGs, RG3, RG9 and RG13, may be unique to Australia. However, more extensive sampling from other countries is required to ascertain whether these three RGs are restricted to Australia. RG8 and RG12A genotypes appear to be infrequently isolated in Australia^14^.

### Progressive spacer deletion in the CRISPR evolution of *S*. Typhimurium

CRISPR arrays appear to evolve by the deletion or duplication of spacer-repeat units or by the rare acquisition of new spacers^18^. The divergence of the clades was associated with progressive losses of spacers (Fig. 1). For example, Clades I and II have lost sp20 and sp21 of CRISPR2 indicating an earlier divergence of Clade II from Clade III and Clade I has lost both sp25 to sp28 of CRISPR1 and sp31 to sp33 of CRISPR2 when Clades I and II diverged from their most recent common ancestor.

Generally, there were few single spacer deletions. Most deletions involved two or more spacers such as deletion of sp2 to sp32 of CRISPR2 in SARA21 that presumably occurred in one event. Many deletions were across lineages or RGs as single deletion events indicative of common ancestry rather than parallel loss (Fig. 1). There were many RG-specific spacer deletions. Sp31 of CRISPR1 and sp37 of CRISPR2 which were only present in Clade V, and sp30 in CRISPR1 which was only present in Clade IV may be more recent acquisitions.

Duplication of spacers can also now be better understood. Some duplications such as sp5, sp11 and sp21 in CRISPR1 arrays and sp14 in CRISPR2 arrays appeared to be rare events as they were only seen in single isolates^14^. Duplication of sp16 in CRISPR1 was only seen in RG9B and duplication of sp28 in CRISPR1 was only seen in some RG2 genotypes. However, duplication of sp15 in CRISPR2 arrays was much more widespread except for RG2, RG6, RG9A, RG12B and RG12C which have only one sp15. Therefore, this duplication may be an ancient event and has only been lost by relatively few genotypes as evolution has progressed.

### Prophages found in the 105* S*. Typhimurium genomes

**Figure 2:**
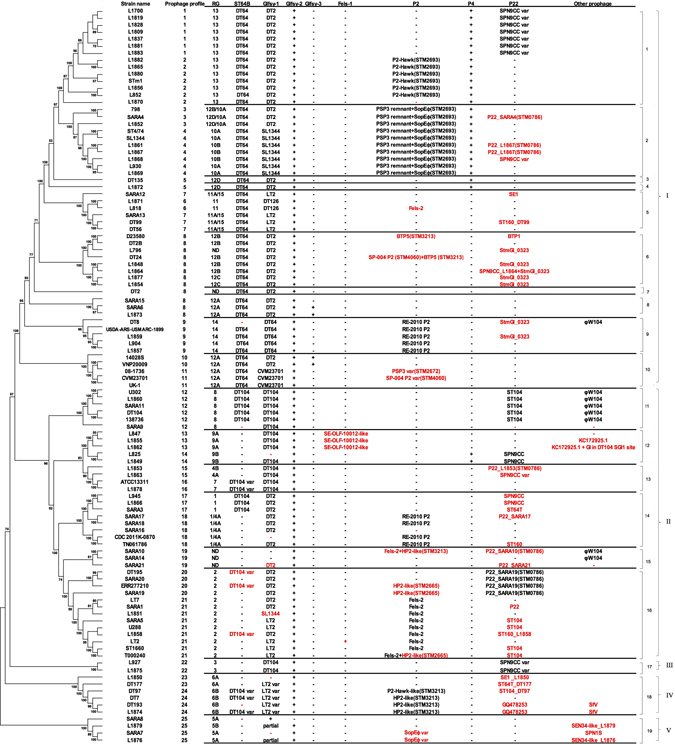
Phylogenetic tree of 105 *S*. Typhimurium strains and distribution of prophages and prophage profiles. “+” and “−” in the prophage profiles indicate the presence and absence of prophage. Additional insertion or deletion of prophages compared with universal patterns are labelled in red. Var is designated after the named phage indicating it is a variant of that phage. The insertion site that differs from the usual position is indicated in brackets. The lineage and clade number are indicated on the right-hand side of the figure.

Our previous studies have shown that much of the genomic diversity within *S*. Typhimurium was due to variation in prophage content^4^. This study extends that observation and presents a fuller characterisation of the *S*. Typhimurium prophages. Prophages in *Salmonella* are categorised into five groups, P27-like, P2-like, lambdoid, P22-like, and T7-like^19^. All except the last group were found in our *S*. Typhimurium strains. We also identified three new variants of HP2-like P2 prophages, several new variants of P22 prophages, including some in a novel insertion site STM0786 (LT2), a novel OLF-SE9-10012-like prophage and other outlier prophages. The prophage distribution is illustrated in Fig. 2.

#### Lambdoid prophages

The lambdoid prophages include Gifsy-1, Gifsy-2, Gifsy-3 and Fels-1. Gifsy-1 was previously subtyped into seven variants, Gifsy-1_LT2_, Gifsy-1_DT104_, Gifsy-1_DT64_, Gifsy-1_SL1344_, Gifsy-1_DT2_, Gifsy-1_CVM23701_ and Gifsy-1_DT126_^14^. Gifsy-1_LT2_ was present in five of the 13 RG2 strains and in four RG11A/15 strains. A variant of Gifsy-1_LT2_ which has three segments different from LT2 occurred in five of the six RG6 strains. Gifsy-1_DT104_ was found in RG3, RG4, RG7 and RG8. Gifsy-1_DT126_ was exclusive to RG11. Gifsy-1_DT64_ was only found in RG14. Gifsy-1_CVM23701_ was exclusive to some RG12A strains. Gifsy-1_SL1344_ was found in RG10 and one strain from RG2. Gifsy-1_DT2_ was distributed widely in many RGs including RG13, RG12, RG1, RG2 and RG1/4A.

Gifsy-2 was found in all strains except SARA8 and is highly conserved with no variants as reported previously^20^. Gifsy-3 was found in SARA6, L1873, 14028 S and VNP20009 with >99% DNA sequence similarity to each other. Fels-1 was only found in LT2.

#### P27-like prophages

The P27-like prophage, ST64B has 2 variants, ST64B_DT64_ and ST64B_DT104_. The ST64B_DT64_ variant was found in all Clade I strains and not in any other clades (although a previous study by Hiley *et al*.^14^ showed that it was present in a minority of RG2A isolates in Clade II) while the ST64B_DT104_ variant was only found in some strains in Clades II and IV. Comparison of these ST64B_DT104_ prophage sequences in RG2, RG7 and RG6B to prototype ST64B_DT104_ in DT104 showed that some strains contained ST64B_DT104_ with varying degrees of coverage (range from 57.4% to 94.5%) (Supplementary Table S4).

#### P2 prophages, P2 variants and P4 prophage

Forty eight strains were found to contain one of the P2 prophages, Fels-2, RE-2010, PSP3, SopEφ, SP-004, 186-type, P2-Hawk and other HP2-like or variants of these. Three strains, DT24, SARA10 and T000240, had two P2 prophages in different locations. All RG10 strains had a remnant PSP3 P2 prophage.

Fels-2 was found in nine of the 13 RG2 strains as well as SARA10 (RG unassigned) and L818 (RG11). RE-2010^21^, a variant of Fels-2, was found in two different lineages RG1/4A and RG14. SopEφ, another Fels-2 variant, always in tandem with a P4 prophage, was found in all RG10 strains as well as the three RG10A hybrids. P4 is a satellite phage using P2 as a helper phage^20^. A SopEφ variant was found in RG5A strains L1876 and SARA7 and was also inserted in the Fels-2 site. The SopEφ variant showed lower similarity to *S*. Typhimurium SopEφ, (Accession No. AY319521, 85%/97% coverage/identity for L1876) than to the SopEφ in *S*. Javiana 10721 (AOZA01000057) with 93%/98% (coverage/identity), indicating that it may have come from another serovar. A variant of SP-004 was found only in CVM23701 and DT24 while a variant of 186-type P2 was found in D23580 (BTP5) and DT24.

HP2 phage (NC_003315) was first found in *Haemophilus influenzae* ^22^, and a distantly related variant, P2-Hawk, was reported in *S*. Typhimurium strains in tandem with a P4 prophage^17^. In our study, we found 14 HP2-like prophages in various insertion sites. Six of these were identified as P2-Hawk and were all in RG13 strains.They were almost identical, differing by no more than five SNPs from each other and always in tandem with the P4 phage. A comparative analysis showed that a core genome of 10 kb was shared by HP2-like P2 prophages, but only 2.6 kb was shared by all the genomes of HP2-like, P2, Fels-2, RE-2010 and SopEΦ (Table S5). Phylogenetic analysis of the core genome of HP2-like P2 prophages showed that these prophages were divided into three variants all with considerable divergence from HP2 phage (NC_003315) (Figure S2). One variant was found in RG6B strains L1874, DT7 and DT193, inserted at the LT2 gene STM3213 which is the same insertion site as for BTP5, a coliphage 186-type P2 phage in strain D23580. This phage has also been found in strains of serovar Enteritidis and Newport with 99%/99% coverage/identity. The SARA10 HP2-like phage (RG unassigned) was also related to this variant but was closer to that found in serovar Heidelberg SL476. Strain DT97 (RG6B) had a variant which clustered with P2-Hawk but inserts at STM3213 instead of at STM2693. Another variant, which inserted at the LT2 gene STM2665 site, was found in ERR277210, T000240 and SARA19 all in RG2. P4 was present independently of P2 in RG9B, RG12D and more than half of the RG13 isolates.

#### P22 prophages and P22 variants

Forty six strains were found to contain a P22-like prophage or one of its variants. The P22-like prophages that are located in the STM0323 site have seven publicly available variants including P22 (NC_002371), SE-1 (DQ003260), SPN9CC (JF900176), ST160 (GU573886), ST104 (AAF75053), ST64T (AY052766) and BTP1 (D23580). We first used PHAST to assign the 46 P22-like prophages into the above categories. P22 (NC_002371) or its variant was found in SARA1, SARA17, and SARA21. There were other variants of P22 (NC_002371) in SARA4, L1861, L1867 (RG10), L1853 (RG4A), SARA10 (RG unassigned) and five strains from RG2 (SARA1, DT195, ERR277210, SARA19 and SARA20) but these were located in a novel insertion site, STM0786. SE-1-like prophages were found in SARA12 and L1850 with 100%/99% and 82%/99% coverage/identity, respectively. SPN9CC or one of its variants was found in 16 strains spread across six RGs. ST104 and one of its variants in DT97 were found in another 11 strains. ST160 was found in three strains, of which L1858 and DT99 each had a variant. Additionally, two strains had ST64T and strain D23580 had the BTP1 prophage.

We analysed the genetic diversity within P22 prophages. A phylogenetic tree of core genome sequences showed that the P22-related SPN9CC-like prophages could be divided into three variants (Figure S3). The first variant had only a few SNPs different from SPN9CC and was in RG1 strains L945 and L1866. The second variant contained a 1,213 bp deletion of the *nin* gene region in the position of 15268–16480 in phage SPN9CC (JF900176) and was in 13 strains from RG12, RG13 and RG10. The third variant had six unique sequences and was present only in L1864 from RG12B.

There was considerable diversity of gene content within the P22-like prophages. To better delineate the gene content variation of various P22-like prophages, we analysed the pan-genome of the 46 P22-like prophages found in this study as well as the genomes of seven publicly available P22 and its variants and obtained a 100 kb pan-genome. The pan-genome consisted of 193 DNA fragments, most of which were only shared by some prophage genomes (Supplementary Table S6). Only 11 fragments of 6.5 kb were shared by all, of which 3 kb belonged to capsid assembly genes and scaffold genes, indicating that the capsid assembly and scaffold genes can be conserved among the different P22-like branches. Based on the presence/absence of genes of the pan-genome (Supplementary Figure S4), SE-1, ST64T, ST160 and their variants were grouped into the same cluster, as were the ST104-like prophages and the SPN9CC-like prophages. P22-like prophages showed high genomic diversity as many variants are not closely related to any other P22 variants. Our pan-genome analysis also showed that some P22-like prophages were incorrectly categorised by PHAST. For example, a P22-like prophage in DT177 was initially identified as ST64T by PHAST but the pan-genome analysis showed that it was closer to SE1 with which 19 genes were shared.

We further explored the evolution of P22-like prophages by comparing their sequences with those from *E*. *coli* and other *Salmonella* serovars (Supplementary Table S6 and Supplementary Figure S4). The composition of these prophage genomes was mosaic with sequences from different sources, indicating frequent exchanges of DNA between subgroups of the P22-like phages as well as from *E*. *coli* and other *Salmonella* serovars. The majority of the fragments (137 out of 193) had high similarity to prophages in multiple serovars, while a few only had high similarity to one or two *Salmonella* serovars, including serovars closely related to *S*. Typhimurium such as *S*. Heidelberg, and more distantly related serovars like *S*. Newport and *S*. Paratyphi A. Twenty-four fragments had similarity to *E*. *coli* prophages, while 12 fragments were only found in *S*. Typhimurium prophages. Thus, there has been a considerable exchange of genetic information among diverging P22-like phage lineages, and the exchange appears to be randomly distributed throughout their genomes.

There was considerable sequence diversity for the P22-like prophages located at the STM0786 insertion site. The integrase for these prophages had 68% identity with that from *Enterobacteria* phage HK106 NC_019768. The P22 variant in SARA10 had 66% coverage/99% identity with P22 (NC_002371) but 99% coverage/99% identity with Paratyphi B ATCC 8759 (AOYE01000028.1). SARA4 which was a RG12D/10A hybrid had another variant with 50% coverage/99% identity with P22 (NC_002371). Another prophage, P22_SARA19, was found in SARA19, DT195, ERR277210 and SARA20 but also with considerable sequence divergence from other P22 variants (Table S7). Another variant, P22_L1867, was found in L1861 and L1867 belonging to RG10B. Strain L1853 had a P22 variant which had greater sequence coverage with BTP1 from D23580 (39% with 99% identity) than with P22 (NC_002371) (34% and 99% identity) and even less coverage with the other STM0786 prophages.

#### Other prophages

Five outlier prophages, SPN1S, OLF-SE9-10012-like prophage, a SfV-like prophage, φW104^23^, and SEN34-like which do not belong to any of the known *S*. Typhimurium prophage groups, were found. A SPN1S variant was found in SARA7 which was inserted at the same site (STM2510) as the SPN1S in *S*. Heidelberg strain 12-4374 (CP012924.1). A OLF-SE9-10012-like prophage of 36 kb was found to be inserted at the Fels-1 insertion site in RG9A strains L847, L1855 and L1862. A *Shigella* SfV-like prophage of 45 kb, which was inserted at STM4243, was found in RG6B strains DT193 and L1874. φW104 (belonging to the family Podoviridae) was detected in all but one RG8 strain and a variant of φW104 was found in SARA10 and SARA14 (unassigned RG) and DT8 (RG14). A 42 kb SEN34-like prophage (belonging to the family Podoviridae) in the STM2067 insertion site was found in strains L1876 and L1879.

### Prophage profiles and correlation with core genome and CRISPR evolution

Based on the distribution of ST64B, Gifsy-1, Gifsy-3, Fels-1, P2, P4, P22 and φW104 prophages found in this study, the 105 *S*. Typhimurium strains were classified into 25 prophage profiles (Table 3). Each profile consisted of a set of shared prophages. Within these profiles other prophages may be variably present as shown in Fig. 2. There was a considerable level of correlation between prophage profile and lineages determined by genomic typing (Fig. 2). Prophage profiles, to a certain extent, also reflected the compositions of CRISPR arrays as the strains in many RGs or subsets within RGs have the same phage profile. Since the CRISPR-cas system is possibly involved in defence against phage invasion we investigated whether there is a link between the loss of spacers and gain of a phage. Loss of one or more spacers within an RG could provide a mechanism for phage invasion. A study in *Cronobacter sakazakii* showed that the CRISPR-cas system is active in that species where clinical strains carried few CRISPR spacers and had more phages, suggesting that gaining more prophage by clinical strains may offer an advantage to the host in survival and pathogenicity^24^. However, we did not find any loss of spacers that match the phage genome in corresponding RGs in this study. Shariat *et al*.^11^ have reported that the CRISPR-Cas systems in *Salmonella* are no longer active, arguing against a role in modulating phage invasion in *S*. Typhimurium in recent evolutionary history. Nevertheless, considering that some prophages carry virulence genes (see below), phages may have acted as a driving force in the evolution of *S*. Typhimurium, regardless of the mechanisms of phage acquisition^25^ ^, 26^.

**Table 3:** Prophage profiles for various strain clusters.

RG	Prophage profile number	ST64B	Gifsy-1	Gifsy-2	Gifsy-3	Fels-2	P22 var	Other P2	P4	Other
13	1	DT64	DT2	+	−	−	SPN9CC var	−	+	−
13	2	DT64	DT2	+	−	−	−	P2-Hawk STM2693	+	−
12D(B)/10A	3	DT64	DT2	+	−	SopEφ	+/−	PSP3 remnant STM2693	+	−
10A, B	4	DT64	SL1344	+	−	SopEφ	+(RG10B)/−	PSP3 remnant STM2693	+	−
12D	5	DT64	DT2	+	−	−	−	−	+	−
11	6	DT64	DT126	+	−	−	−	−	−	−
11A/15	7	DT64	LT2	+	−	−	+/−	−	−	−
12A, B, C	8	DT64	DT2	+	−	−	+/−	+/−	−	−
14	9	DT64/−	DT64	+	−	RE-2010 P2	+/−	−	−	−
12A	10	DT64	DT2	+	+	−	−	−	−	−
12A	11	DT64	CVM23701	+	−	−	−	+/−	−	−
8	12	DT104	DT104	+	−	−	ST104	−	−	φW104/−
9A	13	−	DT104	+	−	−	−	−	−	SE-OLF-10012-like
9B	14	−	DT104	+	−	−	SPN9CC	−	+	−
4	15	−	DT104	+	−	−	+	−	−	−
7	16	DT104 var	DT104	+	−	−	−	−	−	−
1	17	DT104	DT2	+	−	−	+	−	−	−
1/4A	18	−	DT2	+	−	RE-2010 P2	+/−	−	−	−
ND	19	−	−	+	−	−	+/−	+/−	−	φW104/−
2	20	DT104 var/−	DT2	+	−	−	P22_SARA19 STM0786	+/−	−	−
2	21	DT104 var/−	LT2/SL1344	+	−	Fels-2	+/−	−	−	−
3	22	−	DT104	+	−	−	SPN9CC var	−	−	−
6A	23	−	LT2 var	+	−	−	+	−	−	−
6B	24	DT104 var/−	DT104	+	−	−	+ or GQ478263	HP2-like STM3213	−	−
5	25	−	−	−	−	−	−	−	−	−

### Prophages in *S*. Typhimurium genomes with similarity to prophages from other serovars and/or other species

The SPN1S variant had some additional genes which shared identity with phage SPC32N or the sequence from *Klebsiella pneumoniae* (Supplementary Table S8), indicating it was a hybrid prophage. The OLF-SE9-10012-like prophage in RG9A strains showed the highest similarity to a prophage in *S*. Enteritidis OLF-SE9-10012 (CP009091.1) with 70%/99% coverage/identity (Supplementary Table S9). This prophage was an unclassified member of the Myoviridae not related to P2 and with little similarity to other members of the Myoviridae family. Related prophages were also found in *S*. Muenchen BAA1674 (AOYT01000011.1), *S*. *bongori* N268-08 (CP006608.1), *S*. Hartford str 2012K-0272 (ARYS01000018) and *S*. Bovismorbificans 3114 (HF969015.2) as well as in multiple strains of *S*. Weltevreden and *S*. Bareilly. Close to the 3’ end of the prophage was the location of a variant form of the *sopE* gene (AF043239) which had been previously identified as a feature of all RG9A genotypes by PCR^14^.

The SfV-like prophage in two RG6B strains showed considerable divergence from SfV (AF339141) (Supplementary Table S10). It had a mosaic composition with some genes encoding hypothetical proteins coming from other species or other *Salmonella* serovars. These genes were inserted into different positions of the SfV-like prophage genome, indicating that multiple genetic exchanges have occurred (Figure S5). The SfV-like prophage lacked a 6 kb region present in SfV which carries the *gtr* genes for serotype conversion^27^. Additionally, the 5′ end region encoding phage packaging and structure, and right-hand side regions encoding replication and regulation, were partially replaced by sequences from other serovars with unknown functions. The SEN34-like prophage shared only 20% of its genome with phage SEN34 (KT630649.1). A SEN34-like prophage was also found in serovar Weltevreden 1655 (CP014996.1) and Paratyphi B SPB7 (NC_010102). L1879 had a 43.5 kb sequence in the same insertion site that had only 14% coverage with SEN34. The remaining sequence was found in a number of *S*. Typhimurium strains and in other *Salmonella* serovars.

### Distribution of virulence genes carried on prophages

*S*. Typhimurium prophages may carry genes that enhance the virulence of the bacterial host^28^ thus the distribution of these genes deserves closer attention (Supplementary Table S11). The four virulence genes carried on Gifsy-1, *gipA*, *gogB*, *gogD*, and *gogA*, were present in nearly all except seven strains from four different RGs. Interestingly, these genes were mostly absent in the earliest diverged lineage (RG5). The *artAB* genes on Gifsy-1_DT104_ initially found in epidemic DT104 strains^29^ were found in other strains in six RGs. Similarly, the seven virulence genes carried by Gifsy-2 were present in most strains. However, *sopE* and *sspH1* carried by a P2 and Gifsy-3 respectively were variably present in two different branches, while *nanH* carried by Fels-1 was only present in one strain (LT2). Some of these genes play a key role in virulence. Specifically, *SopE* activates RHO GTPases that lead to modification of cytoskeleton of the host cell for invasion and also induce caspase 1 to provoke inflammation^30^. *SopE* can also induce the production of nitrate by host cell so that *Salmonella* can use nitrate respiration in the gut^31^, which enhances the survival of *S*. Typhimurium inside the host and competition with gut microbiota. *SseK*, a T3SS effector, encoded on ST64B and shown to play a role in the inhibition of NF-κB activation^32, 33^, was found in five RGs. Some P22 prophages carry *gtrABC* genes which encode glucosyltransferases that glycosylate the galactose residues of the somatic O-antigen in *S*. Typhimurium^34^. Modification of the O-antigen may help evade host induced immunity. The distribution of the *gtrABC* carrying P22 prophages was random across the genome tree with only 3 RGs fully carrying these prophages. The variable carriage of these prophages and virulence genes suggest that *S*. Typhimurium strains can significantly differ in their pathogenic potential.

### Genomic islands

A novel genomic island inserted in the STM0323 (*thrW* tRNA) site was found and named as StmGI_0323. It was present in some strains of RG12B and RG12C including L1848, L1854, L1864 and L1877 as well as L796. In L1864, StmGI_0323 occurred in tandem with a SPN9CC-related P22 prophage. The genomic island was also found in DT8 and L1859 in RG14. StmGI_0323 encodes 14 open reading frames (ORFs) (Table 4) and is clearly of plasmid origin as the majority of the ORFs, four of which encode conjugal transfer proteins, shared high similarity to *E*. *coli* plasmid sequences (>99% at protein level). It was noteworthy that another unrelated genomic island GQ478253 was inserted at this site in RG6B strains L1874 and DT193.

**Table 4:** Detailed identity comparison of each gene in genomic island StmGI_0323.

Open reading frame no.	Product/size (aa)	Best matches (BLASTp)*	Identity/coverage (%)
ORF 1	Hypothetical protein (470)	*E*. *coli* UMEA 3318-1	100/100
		*E*.*coli* DORA_B_14	100/100
ORF 2	Hypothetical protein (149)	*Cronobacter condimenti* 1330	99/99
		*E*.*coli* DORA_B_14	98/99
ORF 3	Site-specific recombinase (202)	*E*. *coli* ( > 10)	100/100
		*E*.*coli* DORA_B_14	99/99
ORF 4	Hypothetical protein (366)	*E*.*coli* DORA_B_14	99/99
		*K*. *pneumoniae* B1	99/98
ORF 5	HigA (antitoxin to HigB)	*E*.*coli* DORA_B_14	100/100
		*E*. *coli* (5), *Enterobacter* (2)	98/98
ORF 6	Hypothetical protein (97)	*E*. *coli* D6-117.29	100/100
ORF 7	TraJ IncP-type conjugal transfer protein (121)	*E*. *coli* 907357	98/100
		*E*.*coli* DORA_B_14	98/100
ORF 8	TrbJ IncP-type conjugal transfer protein (264)	*E*. *coli* (3)	99/99
ORF 9	Hypothetical protein (57)	*E*. *coli* (4)	96/96
		*Enterobacteriaceae bacterium* FGI 57	93/94
ORF 10	TrbL IncP-type conjugal transfer protein (453)	*E*. *aerogenes* GN02286	98/99
		*E*. *coli* KTE171	98/98
ORF 11	Hypothetical protein (70)	*E*. *coli* (2), *Enterobacter* (2)	100/100
ORF 12	Hypothetical protein	*E*. *coli* (5)	100/100
		*Enterobacteriaceae* bacterium FGI 57	99/98
ORF 13	Putative membrane protein	*E*. *aerogenes* GN02286	100/100
		*E*. *coli* (4)	99/99
ORF 14	Shufflon-specific DNA recombinase	*E*. *aerogenes* GN02286	99/99

*Numbers in brackets indicate the number of strains.

## Conclusions

This study compared the genomic diversity of Australian and international strains of *S*. Typhimurium. The size of core genome in our set was slightly smaller than in previous reports, indicating that we have derived a stable core genome. The 105 strains could be divided into five clades and 19 lineages based on core genome variation. The strains represented 14 different RGs and the RGs primarily derived from CRISPR array composition correlated well with the lineages determined by core genomic typing. Previous studies also found CRISPR composition is correlated with genomic relationship in other *Salmonella* serovars^35^, suggesting this is a general phenomenon. The accessory genome of *S*. Typhimurium contained a fair proportion of prophage genes. Some prophages were widely present in *S*. Typhimurium while others were sporadic. There was a strong correlation of prophage profiles with lineages and CRISPR profiles. Acquisition of phages may have played an important role in the adaptation and virulence evolution of *S*. Typhimurium. There was high sequence diversity among related prophages with a considerable level of similarity with prophages from other serovars and/or other species, suggesting extensive horizontal gene transfer. Virulence genes such as *sopE* carried by prophages were variably present, indicating variation in pathogenicity among *S*. Typhimurium strains. These findings have extended our understanding of the genomic diversity and core genome evolution of *S*. Typhimurium, particularly its relationship with CRISPR evolution and prophage variation.

## Materials and Methods

### Bacterial strains and genomic DNA isolation

Thirty-nine human clinical isolates representing CRISPR diversity^14^ collected between 1997 and 2011 were selected for sequencing (Table 2). Thirty-seven isolates in this study had been referred by the laboratory of Queensland Department of Health, Forensic & Scientific Services in Brisbane, Australia. Two other isolates were obtained from the UK. The phenol/chloroform method was used to extract genomic DNA from each strain as described previously^36^. Sixty-six publicly available genomes were also used as shown in Supplementary Table S2. The plasmid and antibiotic resistance genes among the 105 strains were also analysed (Supplementary text).

### CRISPR profiles

The CRISPR sequences of 36 strains (strain No. from L1849 to L1883) were determined in a previous study^14^. The CRISPR sequences from 35* S*. Typhimurium strains sequenced in our previous studies^4, 6, 16^ were analysed in this study. The CRISPR1 and CRISPR2 sequences in each isolate were amplified using the primer pairs described by Hiley *et al*.^14^. PCR products were sequenced using the Applied Biosystems 3130 sequencer and BigDye Terminator v3.1 Cycle Sequencing Kit. ChromasPro was used to analyse the sequences. For 34 public genomes, the CRISPR finder program (http://crispr.u-psud.fr/) was used to locate the regular repeats and the intervening spacer sequences. Results were represented as filled rectangular blocks for ‘spacer present’ or an X for ‘spacer absent’ in the same order as for *S*. Typhimurium spacers in Table 6 in Fabre *et al*.^8^ (Supplementary Table S2).

### Genome sequencing, *de novo* assembly and identification of Single nucleotide polymorphisms (SNPs)

Genomic DNA was sequenced using the Illumina Genome Analyzer (Illumina) with 250 bp paired end sequencing. Contigs were *de novo* assembled using the Velvet version 1.0.8 and VelvetOptimiser^37^. Large scaffolds and short contigs generated by Velvet were aligned to the *S*. Typhimurium LT2 genome (NC_003197) using progressiveMauve version 2.3.1^38^. RAST was used to annotate the sequences from each NGS genome^39^. The number of coding sequences in the genomes was predicted based on RAST. For draft genomes, SNP calling was performed by Samtools (version 0.1.19) and followed the previously described criteria^6^. A custom script was used to determine whether a SNP in the genic region is synonymous SNP (sSNP) or non-synonymous SNP (nsSNP). For the complete genome, SNPs were determined by using the NUCmer program in the MUMmer package version 3.0^40^.

### Prophage analysis

The presence of prophages from the sequenced strains was screened using PHAST^41^. The prophages were confirmed by searching for integrase from annotated genomes. We subtyped the ST64B prophages and Gifsy-1 prophages into two and seven variants, respectively. ST64B prophage has two variants: ST64B_DT104_ and ST64B_DT64_. The sequence of ST64B_DT64_ (AY055382) and the sequence of ST64B_DT104_ obtained from DT104 (NC_022569) were used as reference sequences to confirm the variants in our studied strains. Gifsy-1 prophages were subtyped as either Gifsy-1_LT2_, Gifsy-1_DT104_, Gifsy-1_DT126_, Gifsy-1_SL1344_, Gifsy-1_DT2_, Gifsy-1_CVM23701_ or Gifsy-1_DT64_ based on the unique sequences among these variants as defined previously^14^. The core genome content of P2 prophage and HP2-like group of P2 prophage were obtained by analysing common shared regions of P2 prophages and HP2-like P2 prophages using progressiveMauve version 2.3.1, respectively^38^.

### Phylogenetic analysis

Based on *S*. Typhimurium core genome SNPs we defined previously^4^ and core genome content of P2 prophage identified in this study, phylogenetic trees were constructed using the Minimum Evolution algorithms in MEGA 5.0 for 105 *S*. Typhimurium genomes and 15 P2 prophage genomes, respectively^42^. Bootstrap analysis was performed with 1,000 replicates. Based on the presence and absence of DNA segments in the P22 pan-genome, a UPGMA tree was constructed using the web-server DendroUPGMA (http://genomes.urv.cat/UPGMA).

### Sequence data accession number

The raw sequencing data were submitted to GenBank (NCBI) under the BioProject No. PRJNA355598.

## Electronic supplementary material

Supplementary Tables

supplementary info
