# Supplementary material for: Comparative genomics of Australian and international isolates of Salmonella Typhimurium: correlation of core genome evolution with CRISPR and prophage profiles
Source: Sci Rep. 2017 Aug 29;7:9733. doi: 10.1038/s41598-017-06079-1 (PMC5575072; doi:10.1038/s41598-017-06079-1)
Supplement: Supplementary file 2 — supplementary info [file 41598_2017_6079_MOESM2_ESM.pdf]

Supplementary Material

Comparative genomics of Australian and international isolates of *Salmonella* Typhimurium: correlation of core genome evolution with CRISPR and prophage profiles

Songzhe Fu[1], Lester Hiley [2], Sophie Octavia[1], Mark M. Tanaka[1], Vitali Sintchenko[3,4], Ruiting Lan[1]†

[1]School of Biotechnology and Biomolecular Sciences, University of New South Wales, Sydney, New South Wales, Australia

[2] Public Health Microbiology Laboratory, Forensic and Scientific Services, Queensland Department of Health, Brisbane, Queensland, Australia

[3]Marie Bashir Institute for Infectious Diseases and Biosecurity, University of Sydney, New South Wales, Australia

[4]Centre for Infectious Diseases and Microbiology–Public Health, Institute of Clinical Pathology and Medical Research, Westmead Hospital, New South Wales, Australia

†To whom correspondence should be addressed. E-mail: r.lan@unsw.edu.au

Supplementary text

Plasmids and antibiotic resistance

We further analysed the distribution of the plasmid and antibiotic resistance genes among the 105 strians. Antimicrobial resistance genes were identified using Resfinder [1]. The contigs that were not aligned with LT2 were identified by progressiveMauve. To determine the homologues and functions of the unaligned sequences, contigs were searched against the GenBank non-redundant nucleotide database by using BLASTn [2]. To determine the presence of pSLT from the 39 strains sequenced, reads and contigs were mapped onto the pSLT sequence in LT2 (NC_003277). There were three common plasmids found in multiple strains including pSLT, pSH1148_107 and pRSF1010_SL1344. In total, 80 out of 105 *S.* Typhimurium strains carried the 90 Kb virulence plasmid, pSLT. Interestingly, the pSLT was absent in some lineages. Strains typed as RG7, RG1 and RG1/4A, RG6 (except for DT193) and RG5 did not possess this plasmid. Besides, pSLT was also absent in L927, USDA-ARS-USMARC-1899, D23580 and 08-1736. However, other plasmids were more likely to be randomly distributed (Table S13). pSH1148_107-like plasmid was found in SL1344, ST4/74, L1874, SARA9 and SARA10, which is a 110 Kb plasmid in L1874 with 84% and 85% DNA sequence similarities, to the *S.* Heidelberg plasmid pCFSAN002069_01 and *S.* Heidelberg plasmid pSH1148_107, respectively. We therefore named it as p1874. SL1344, ST4/74, T000240, L1868 and L1851 commonly had a 9 Kb pRSF1010_SL1344 plasmid.

Some uncommon plasmids were also found in our sequenced genomes. L1879 had a 101 Kb plasmid with *blaCMY-2* (Beta-Kb lactam resistance) which had 99% similarity with *S*. Newport plasmid pCVM22462 (CP009566). Another 101 Kb plasmid with 99% DNA sequence similarities to *Klebsiella oxytoca* strain CAV1015 plasmid pKPC_UVA02 (CP009466) and a 36 Kb plasmid pSTY3-1898 (CP014974) were also identified in L1879. L825 had an additional 40 kb plasmid, namely pL825-2, which has 83% similarity with *S*. Enteritidis plasmid pCFSAN000111_01 (CP007599). DT97 had a 108 kb plasmid with 78% and 94% DNA sequence similarities with pSTM7 (KF290377) and pC49-108 (KJ484638), respectively. Apart from pSLT, ERR277210 also had a 70 Kb plasmid with 80% DNA sequence similarities with pEC3II_2 (KU932023). L1874 had a 4.2 Kb *Escherichia coli* plasmid pV004-b (LC056155). L945 had a 108 Kb plasmid with high homology to both plasmid pHCM2 and phage SSU5[3]. L1858 had a 3 Kb *Escherichia coli* plasmid pEC904(AY589570). L825 has a 4.3 kb *S*. Heidelberg plasmid pSA01AB09084001_4 (CP016532).

Overall, antibiotic resistance genes were randomly distributed among different sub-lineages except for DT97, L1874 and DT193 in RG6B, T000240 and ST1660/06 from RG2 and 138736, DT104, L1860 and U302 in RG8 which shared the similar antibiotic resistance patterns. Antibiotic resistance genes were present in the chromosome of 138736, DT104, L1860 and U302 from RG8, which located in the SG1 genomic island. T000240 and ST1660/06 from RG2 also contain multiple

antibiotic resistance genes in their chromosome as described previously[4].

Antibiotic resistance genes were found in the plasmids of T000240, L1851, L1858, L1860, L1862, L1868, L1874, DT97, DT193, SARA9, SARA10 and L1879 (Table S12). L1851 contains *strA* and *strB* (streptomycin resistance), *bla*$_{\text{TEM-1B}}$ (beta-lactam resistance) and *sul2* (sulphonamide resistance) in its pSLT, while L1858 also contains aac(3)-IV and *bla*$_{\text{TEM-1B}}$ in its plasmid, both of which come from RG1. *sul1* (sulphonamide resistance) and *aadA1* (streptomycin resistance) were found on the pSLT of L1868. L825 contains *strA and strB, bla*$_{\text{TEM-1B}}$ and *sul2* in its pSLT. In L1862, we also found an 28 Kb IncI plasmid with *bla*$_{\text{TEM-1B}}$ and *aadA1*, which inserted into the insertion site of SG1 genome island in DT104 (HF937208.1). In strain L1874, *sul3* (sulphonamide resistance), *bla*$_{\text{TEM-1B}}$, *aadA1* resistance gene and *cmlA1(*chloramphenicol resistance) were found in p1874, while *tetB* (tetracycline resistance) was found on its chromosome. *strA and strB*, *bla*$_{\text{TEM-1B}}$, *dfrA1* (trimethoprim resistance) and *sul2* were found in pSTM7 in DT97, while DT193 has a pSRC27-H multiple antibiotic resistance region (HQ84094) in its chromosome, which contains *strA* and *strB, bla*$_{\text{TEM-1B}}$ and *sul2.*

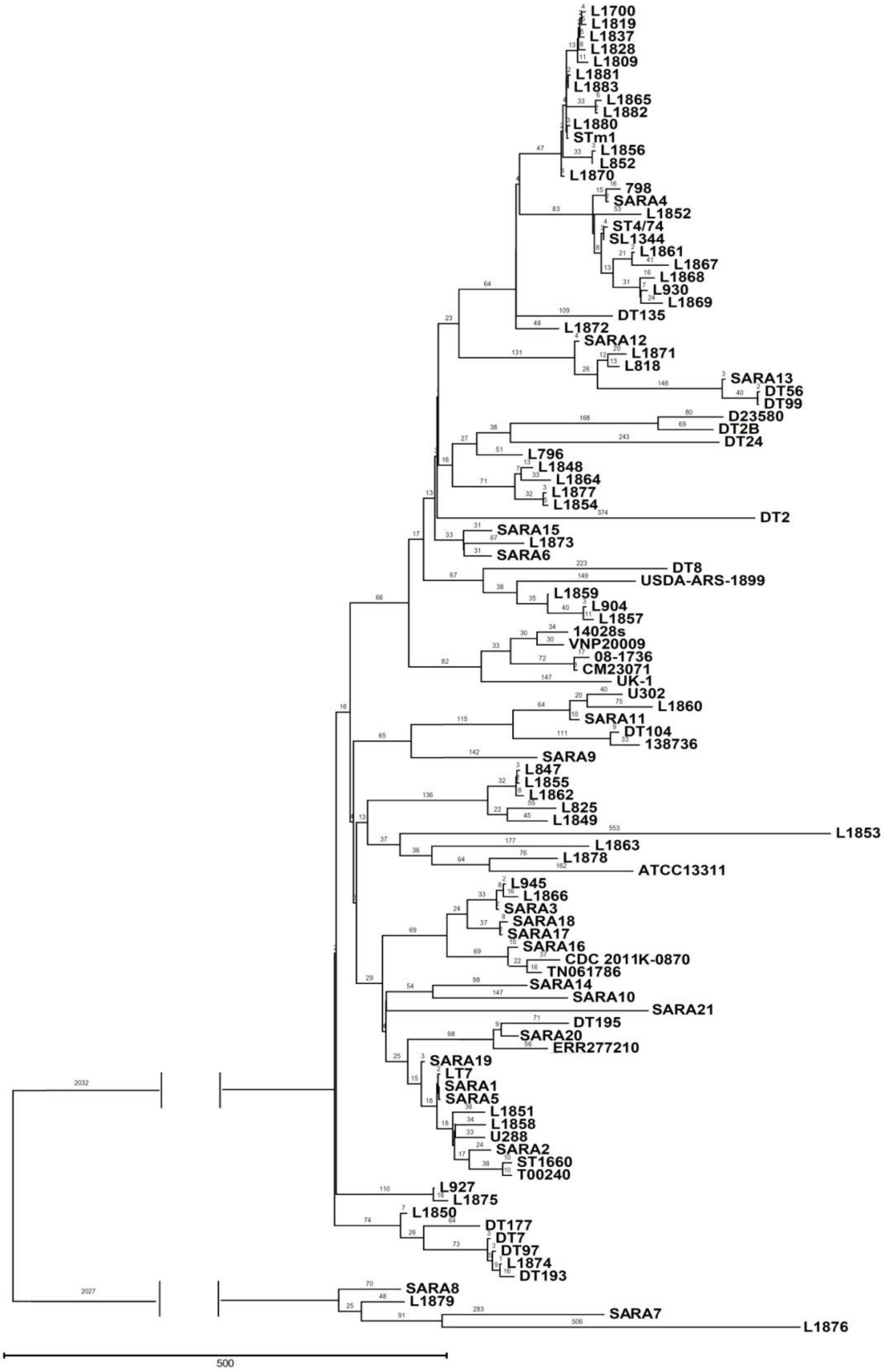

Figure S1 Phylogenetic tree of 105 *S.* Typhimurium strains based on their SNPs obtained from *S*. Typhimurium core genome. The minimum evolution method was used to infer evolutionary relationships of the isolates. The SNPs supporting each branch are shown next to the branches. Scale bar indicates the number of SNPs.

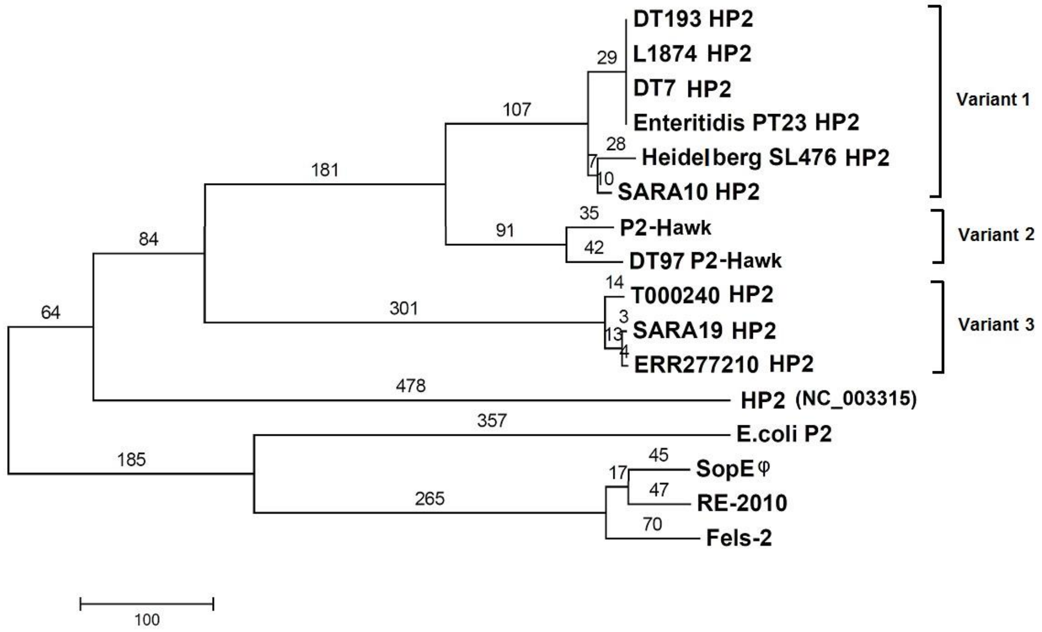

Figure S2 Phylogenetic tree of core genome sequence of P2 phage. The minimum evolution method was used to infer evolutionary relationships of the P2 phages. The SNPs supporting each branch are shown next to the branches. Scale bar indicates the number of SNPs.

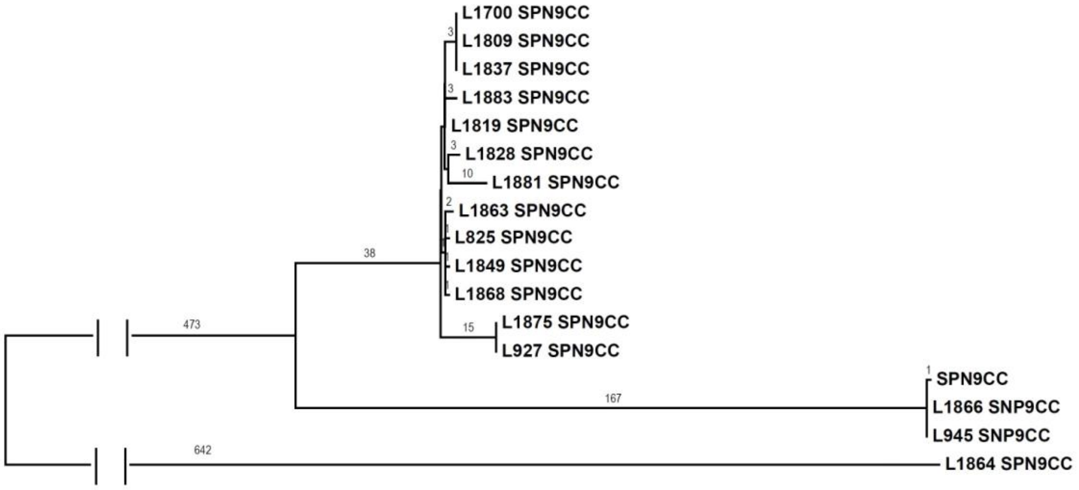

Figure S3 Phylogenetic tree of core genome sequence of SPN9CC phage found in this study. The minimum evolution method was used to infer evolutionary relationships of the SPN9CC phages. The SNPs supporting each branch are shown next to the branches.

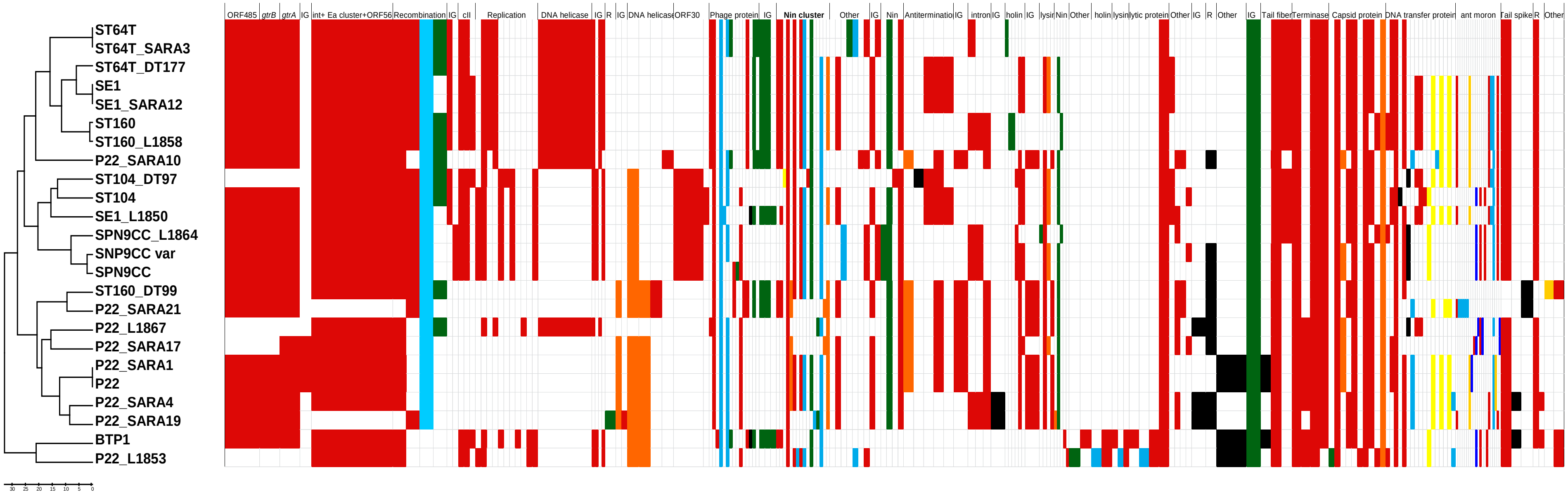

Figure S4 Summary of the phylogenetic relationships among the sequences of 24 P22-like prophages and their homology with other serovars or *Escherichia coli*. Left:The UPGMA tree based on the presence and absence of DNA fragments in P22 pan genome was inferred using DENOUPGMA. The scale bar indicates the number of difference of DNA fragments. The tree is drawn to scale, with branch lengths in the same units as those of the distances used to infer the UPGMA tree. Right: the sequence similarity of 24 P22-like phages and their homology with other serovars or *E. coli*. The cut-off value of sequence similarity is 0.9. Phage P22 gene function is shown at the top in the order in which they are located on the phage pan-genome (not drawn to scale).The gene function was annotated by RAST (except for the use of P22 phage gene names for ORF485, *gtrB*, *gtrA*, int, ORF56, Ea cluster, ORF30 and intergenic region (IG)). The different colours of the phage names indicate the genus of their host as follows: blue: only found in *Escherichia coli*; black: not found in any other serovars or *E.coli* but only in Typhimurium; yellow: Dublin/Choleraesuis; orange: Heiderberg; purple: Newport/Paratyphi A; green: only found in one serovar except for Heidelberg;

Red: found in more than three serovars. Black vertical lines indicate protein/gene boundaries.

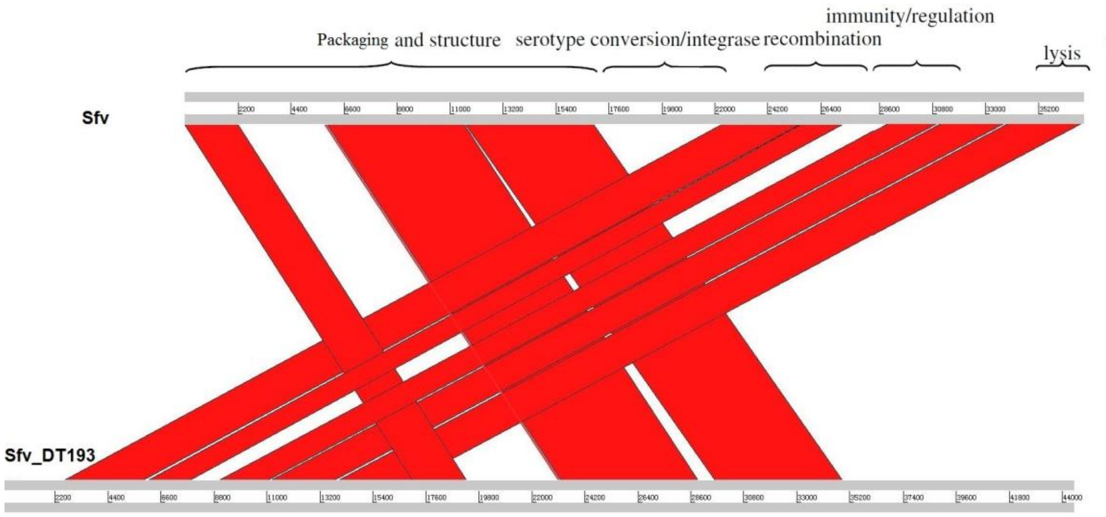

Figure S5 The genome comparison of *Shigella* phage SfV and its variant in DT193. The nucleotide matches between the phage SfV and its variant in DT193 was visualised by Artemis Comparison Tool (ACT) (http://www.sanger.ac.uk/Software/ACT/). The red bars represent individual sequence matches between the DNA lines with each other.

Reference

1 Zankari, E. *et al.* Identification of acquired antimicrobial resistance genes. *The Journal of antimicrobial chemotherapy* **67**, 2640-2644, doi:10.1093/jac/dks261 (2012).

2 Altschul, S. F., Gish, W., Miller, W., Myers, E. W. & Lipman, D. J. Basic local alignment search tool. *J Mol Biol* **215**, 403-410, doi:10.1016/S0022-2836(05)80360-2 (1990).

3 Octavia, S., Sara, J. & Lan, R. Characterization of a large novel phage-like plasmid in *Salmonella enterica* serovar Typhimurium. *Fems Microbiol Lett* **362**, fnv044, doi:10.1093/femsle/fnv044 (2015).

4 Izumiya, H. *et al.* Whole-genome analysis of *Salmonella enterica* serovar Typhimurium T000240 reveals the acquisition of a genomic island involved in multidrug resistance via IS1 derivatives on the chromosome. *Antimicrob Agents Chemother* **55**, 623-630, doi:10.1128/AAC.01215-10 (2011).
